# A theoretical analysis of the barriers and facilitators to the implementation of school-based physical activity policies in Canada: a mixed methods scoping review

**DOI:** 10.1186/s13012-017-0570-3

**Published:** 2017-03-27

**Authors:** Katie A. Weatherson, Heather L. Gainforth, Mary E. Jung

**Affiliations:** 1 School of Health and Exercise Sciences, Faculty of Health and Social Development, University of British Columbia, Okanagan, ART 360-1147 Research Road, Kelowna, BC V1V 1V7 Canada; 2 School of Health and Exercise Sciences, Faculty of Health and Social Development, University of British Columbia, Okanagan, ART 129-1147 Research Road, Kelowna, BC V1V 1V7 Canada; 3 School of Health and Exercise Sciences, Faculty of Health and Social Development, The University of British Columbia, Okanagan, RHS 119-3333 University Way, Kelowna, BC V1V 1V7 Canada

**Keywords:** Scoping review, School, Physical activity, Policy, Implementation, Barriers, Facilitators, Theoretical Domains Framework

## Abstract

**Background:**

Given the potential impact school-based daily physical activity (DPA) policies can have on the health outcomes of Canadian children, it is surprising that such little research has examined the implementation and student-level effectiveness of these policies, and that even less have used theory to understand the barriers and facilitators affecting uptake of this policy by teachers. This review descriptively summarizes the implementation status, approaches used to implement DPA, and the effectiveness of DPA at increasing the physical activity of children at school. In addition, the Theoretical Domains Framework (TDF) was used to explore the barriers and facilitators to DPA implementation.

**Methods:**

A scoping review of English articles using ERIC, CINAHL, and Google Scholar (2005 to 2016) was conducted. Only studies that evaluated the implementation and/or student-level effectiveness of DPA policies in Canadian elementary schools were included. Only articles that examined DPA implementation barriers and facilitators by teachers, principals, and/or administration were eligible for the TDF analysis. Data on study characteristics and major findings regarding implementation status, implementation approach used, and impact on student’s physical activity were extracted and were summarized descriptively, including study quality indicators. Two coders extracted and categorized implementation barriers and facilitators into TDF domains.

**Results:**

The search resulted in 66 articles being retrieved and 38 being excluded for not meeting the eligibility criteria, leaving 15 eligible for review (10 of which examined barriers and facilitators to implementation from DPA deliverers’ perspective). Eleven of 15 studies examined the Ontario DPA policy, and 2 studies were from both Alberta and British Columbia. Thirteen studies examined implementation, and only two examined effectiveness. DPA implementation status, approaches to delivery, and effectiveness on student’s PA levels are inconsistent across the three provinces. A total of 203 barriers/facilitators were extracted across the ten implementation studies, most of which related to the *environmental context and resources (*ECR; *n* = 86; 37.4%), *beliefs about consequences* (*n* = 41; 17.8%), and *social influences* (*n* = 36; 15.7%) TDF domains.

**Conclusions:**

With the limited research examining the DPA policy in Canada, the current status and approaches used to implement DPA and the student-level effectiveness is not well understood; however, this review revealed that DPA deliverers often report many barriers to DPA implementation. Most importantly, in conducting a TDF-based analysis of the barriers/facilitators affecting implementation, this review provides a theoretical basis by which researchers and policy-makers can design interventions to better target these problems in the future.

**Registration:**

A protocol for this review was not registered.

**Electronic supplementary material:**

The online version of this article (doi:10.1186/s13012-017-0570-3) contains supplementary material, which is available to authorized users.

## Background

Like most children and youth worldwide [1], Canadian children are not meeting the national physical activity guidelines for optimal health [2–4]. To address this problem, the World Health Organization recommends that schools develop policies to increase physical activity among children [5]. In an attempt to help children meet the national recommendations of 60 min of moderate-to-vigorous physical activity (MVPA), three Canadian provinces have adopted daily physical activity (DPA) policies aimed to increase children’s physical activity levels specifically during the school day [6–8]. Although the specific DPA policy requirements for each province varies slightly, they are comparable in that they require elementary schools (and thus teachers, principals, and/or administration) to provide a specific amount of time each day for children to be active during instructional hours of the school day. For example, the Ministry of Education in Ontario mandated their DPA policy in 2005, requiring elementary schools to provide at least 20 min of sustained MVPA as part of the instructional school day for children in grades one to eight [8]. Similar DPA policies were authorized in Alberta and British Columbia in 2005 and 2008, respectively, with the requirement to provide activities that vary in form and intensity for 30 min during the school day [6, 7]. Although DPA policies ultimately aim to change and have an effect on students’ physical activity levels at school, within the context of elementary schools, the implementation of DPA policies require behavior change of the teacher to provide opportunities for children to be active, and the approaches they chose to provide these opportunities is left at their personal or school’s discretion. In this way, the DPA policies potentially affect two different, yet interrelated behaviors (the provision by teachers and the physical activity of students). Therefore, if DPA policies are implemented as intended, teachers, principals, and/or administration will change their provision/implementation behaviors, and students will change their physical activity behaviors.

While there are many examples of policies being adopted to promote the physical activity of children [9, 10], “the adoption of policies is not sufficient to promote greater physical activity: policies are not self-implementing” (p.280) [11]. Implementation is the conversion of policy plans into action [12], and implementation evaluation examines the progress and process of how this occurs and measures the products resulting from the process [13]. There are many individual, environmental, and social-cultural factors that influence the successful implementation of policies at a local level. This is especially true of schools, which are “dynamic, complex, multi-level systems with numerous factors that can influence implementation” (p.274) [14], and the quality of implementation can affect the outcomes of the policy or program [15]. Therefore, studying only the adoption of policies while ignoring the context in which they are implemented is detrimental to understanding *how* and *why* policies are or are not successful. A holistic approach that considers the complex interaction of these factors must be taken into account when considering how physical activity policies are implemented in various school-settings.

Although it has been a decade since the first DPA policy was mandated in Canada, evaluation of its implementation and effectiveness is surprisingly limited [16, 17]. Provincial school policies that have the potential to positively impact the health outcomes of so many Canadian children warrant further investigation as to their current implementation and effectiveness. A recently published review examining the adoption, diffusion, implementation, and impact of DPA policies across Canada rated the strength of each province’s policy based on the language used, the specific time and intensity requirements, and the inclusion of mechanisms for implementation and monitoring [17]. This review highlighted that the implementation of these policies across Canada is inconsistent and suboptimal. Additionally, only one study in BC [18] and two studies in Ontario [19, 20] have examined the effectiveness of DPA policy implementation at increasing children’s PA levels at school, with mixed results. It should be noted, however, that the BC evaluation of DPA examined only the impact of DPA on provision of physical education minutes per week, not on overall physical activity levels at school [18]. These mixed findings further highlight the need to examine the factors that prevent implementation in order to understand why the policy is not having a positive impact on children’s PA levels at school. While the authors of this review thoroughly examined how each policy was conceptualized and adopted by each province, they did not use theoretical principles to review the evaluation pertaining to the implementation and impact of these policies on students’ physical activity at school, important components of understanding the policy process [21]. Additionally, of the articles they included in their review, few of the authors reported explicit use of behavior change theory to guide their original research or analyze the factors affecting the implementation process. Theory is valuable for understanding how a policy is put into practice (i.e., implementation) and in identifying the barriers (i.e., factors preventing implementation) and the facilitators (i.e., factors enhancing implementation) that influence policy implementation, in order to explain the impact these policies have on children’s physical activity levels. There are many factors associated with implementing interventions and policies in real-world settings, which requires behavior change at an individual, organizational, or community level [22]. The implementation of the DPA policy during the school day requires behavior change of the teacher, principals, and/or administration, and thus it is important to examine perceived barriers to implementation from this perspective. While identifying barriers to implementation is a common area of inquiry in implementation research, theory is often not used to guide our understanding of these factors [23], which if addressed would be able to increase systematic uptake and success of these policies. The advantage of conducting a theory-based analysis of the barriers and facilitators affecting the implementation of school-based physical activity policies by teachers is that it provides a framework for comprehensively understanding the relationship between these factors and the mechanisms by which they affect teachers’ behavior. Understanding these connections from a theoretical perspective better helps inform and guide researchers, policy-makers and individuals responsible for delivering such policies on how to develop evidence-based strategies to improve uptake of the policy into practice. Simply identifying barriers that are not linked to theoretical constructs does not provide a strong foundation for intervention development.

One such framework that can allow us to apply theory and comprehensively identify the factors that need to be addressed is the Theoretical Domains Framework (TDF) [24, 25]. The TDF is a suitable framework for retrospectively examining barriers and facilitators. It accounts for the overlapping constructs that exist across behavior change theories and it provides categories called domains by which to more broadly capture the potential range of factors that influence implementation outcomes, thus allowing researchers to better understand policy implementation [25, 26]. It also provides a common language for researchers to classify barriers and facilitators to implementation. The 14 TDF domains include knowledge, skills, memory, attention and decision processes, behavioral regulation, social/professional role and identity, beliefs about capabilities, optimism, beliefs about consequences, intentions, goals, reinforcement, emotion, environmental context and resources, and social influences [22]. The TDF has been used in several reviews to understand barriers and facilitators to a wide variety of behaviors (e.g., patients’ exercise behavior, healthcare professionals’ behaviors in relation to pregnancy weight management) [27, 28]. An examination of the barriers and facilitators to DPA implementation by DPA providers (i.e., teachers, principals, and/or administration) using the TDF will provide a list of the potential modifiable factors to target and allow researchers to create theoretically informed interventions to improve the implementation and effectiveness of this school-based physical activity policy in the future.

### Purpose

The aim of this review was to broadly understand the implementation and effectiveness of the DPA policy in Canadian elementary schools. Specifically, we aimed to examine: (1) the implementation status of DPA in Canada, (2) the implementation approaches used to deliver the DPA policy during the school day, (3) the barriers and facilitators to DPA policy implementation, and (4) the effectiveness of DPA policy implementation at increasing the physical activity of children at school.

## Methods

### Approach

Due to the variety of methods used across a small number of existing evaluations, a systematic review and meta-analysis were not possible. Instead, this mixed methods scoping review, guided by the Arksey and O’Malley framework [29], provides a systematic description and synthesis of data. Scoping reviews are appropriate for summarizing broad, understudied areas and identifying gaps in the literature [29]. In addition, the Theoretical Domain Framework was used to code barriers and facilitators to DPA implementation. The Preferred Reporting Items for Systematic Reviews and Meta-Analyses (PRISMA) criteria guided reporting of the methods and findings (see Additional file 1) [30]. A protocol for this review was not registered.

### Search and screening

**Fig. 1:**
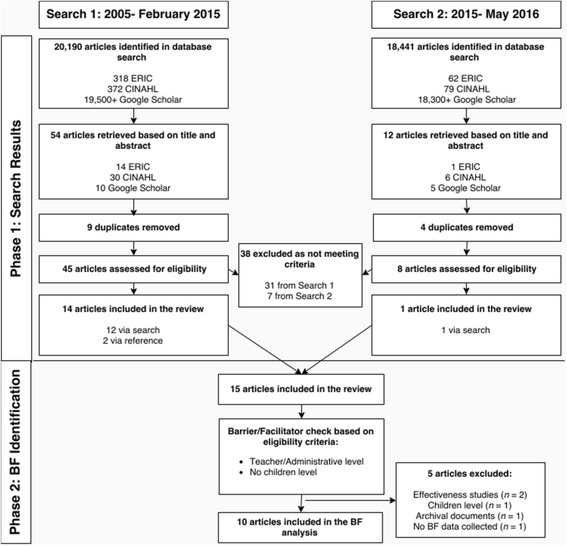
Flow chart of search results and barrier/facilitator (BF) identification. *BF* barrier/facilitator. Search for eligible articles was conducted in Phase 1. Phase 2 involved the identification of articles that examined the barriers and facilitators to implementation

To retrieve research articles and governmental reports on policy evaluation of DPA in Ontario, Alberta and British Columbia, two databases (ERIC, CINAHL) and one search engine (Google Scholar, to identify gray literature), and respective provincial government/education websites were searched in February 2015 for the time period 2005–2015. The same search was conducted again in May 2016 to retrieve additional articles published after the original search. One author executed the searches in consultation with a librarian. The search query was tailored to the specific requirements of each database and broad search terms included: *daily physical activity* OR *physical activity* OR *exercise* AND polic* AND *school*. Additional terms were used in the advanced search option of Google Scholar, to find articles with all of the words: school AND polic* AND Canada, and with at least one of the words: *daily physical activity* OR *physical activity* OR *exercise* AND *qualitative* OR *quantitative.* An a priori decision was made to screen only the first 100 hits (as sorted by relevance by Google Scholar) after considering the time required to screen each hit and because it was believed that further screening was unlikely to yield many more relevant articles. Finally, reference lists of identified articles were examined to retrieve additional eligible articles. One author screened titles and abstracts against eligibility criteria and full texts were retrieved in situations where relevance was uncertain. Each eligible article was read in its entirety to identify studies that examined the barriers and facilitators to DPA implementation. The screening process to obtain the eligible studies is illustrated in Fig. 1. Phase 1 included the search for eligible studies for the overall review, and phase 2 included reviewing the implementation articles for the examination of barriers and/or facilitators.

### Eligibility criteria

Included studies were those that examined any aspect of the implementation or impact of DPA in Canada using qualitative and/or quantitative methods. Government reports were also included in the review. Inclusion criteria for articles and reports were (i) articles written in English, (ii) publication after 2005 (after first provincial policy was mandated), (iii) involved some aspect of DPA policy evaluation (implementation or impact), (iv) applicable to elementary school setting (children aged 5–12 years), and (v) primary research papers. Articles were excluded if they applied only to a secondary school setting (youth aged 13–18 years), as both Alberta and Ontario’s DPA policies do not apply to these students. Unless published dissertations were not included in this review. Articles that only addressed participants’ perspectives or opinions of PA outcomes and did not include formal measurement of DPA (either subjective or objective; i.e., survey, interview, pedometer) were considered implementation articles (not effectiveness). To answer the third aim of this study, implementation articles were examined for the presence of barriers and facilitators, operationalized as any factor, characteristic, view, or belief that either impedes or enables implementation of the DPA policy. For this analysis, eligible articles included those that examined barriers and facilitators from the perspective of teachers, principals, and/or administration.

### Data extraction

The first author extracted the following data from each article: (1) study type and design, (2) participants, (3) methods used to assess implementation and/or PA outcomes, and (4) major findings (process and/or outcome results). For the purposes of study type classification, only student self-reported or objectively measured physical activity was considered an impact measure and classified as an *effectiveness* article. If an article asked teachers, principals, and/or administration to report on children’s physical activity (based on their observation), this study was classified as an *implementation* article. If measured, DPA implementation status (i.e., degree to which DPA was delivered) and approaches used to implement DPA (i.e., methods of DPA delivery) were extracted by the same researcher. Additional information was extracted from each article examining the implementation barriers and/or facilitators, including: (1) data collection method and (2) behaviour change theory used, if applicable. Barrier and facilitator extraction was performed by one researcher, with double extraction occurring across 33% (*n* = 3) of the articles by a research assistant. To identify barriers and facilitators, each article was read in its entirety by both researchers. We distinguished between a barrier and facilitator based on how the authors of each article reported and classified the factor influencing DPA policy implementation. If the authors did not provide this distinction, we used our operationalized definition stated previously. Once identified, each researcher transferred the factor to an excel spreadsheet. For qualitative studies, the barrier/facilitator was recorded in its original format unless only reported by authors in a synthesized format (e.g., according to a themed code). For quantitative studies, individual barriers/facilitators were extracted if ≥50% of respondents agreed that the factor influenced implementation. In other words, a factor was not extracted if >50% of respondents disagreed that the barrier/facilitator was significant. Choosing to extract the barriers and facilitators that were viewed by the majority of respondents as being significant influences to policy implementation allows researchers to provide recommendations for and develop interventions that target these pertinent factors in the future and are hopefully relevant across multiple school contexts. For questionnaire measures with an intermediate category (i.e., Likert-scale questions), the barrier/facilitator was extracted if at least 50% of respondents agreed with the intermediate category (or agreed more strongly; see more extraction details in the comments column of Table 3). If a quantitative study included open-ended questions about implementation barriers or facilitators, the responses were extracted irrespective of how many respondents agreed they were present. Extracted factors from each coder’s excel spreadsheet were compared to assess extraction agreement across the three studies.

### Quality assessment

Although not a requirement in Arksey and O’Malley’s [29] scoping review framework, it has been suggested by others to include an assessment of methodological quality in included studies [31]. Due to the lack of validated quality assessment tools for process evaluations, the adapted version of the criteria described by Naylor and colleagues [32] and originally adapted from Wierenga and colleagues [33] was used (see items and evaluation criteria in Additional file 2). In accordance with Naylor and colleagues [32] past work, items were scored as positive, negative, or not applicable, and studies were classified as strong (>75% positive), moderate (50–75%), or weak (<50%). When an item was not applicable, that item was excluded from the mean score of that study’s rating. One reviewer conducted quality assessments for all implementation articles, with a second rater assessing 33% (*n* = 4) of the articles. Quality assessment agreement was based on overall global ratings not on individual items. For the two studies examining DPA policy’s effectiveness on children’s physical activity [19, 20], the validated quality assessment tool for quantitative studies developed by the Effective Public Health Practice Project (EPHPP) [34] was used (see items and evaluation criteria in Additional file 3). The EPHPP quality assessment tool assigns a strong, moderate, or weak rating to six study components to provide a global quality rating. Strong studies have four or more strong components and no weak components. Moderate studies have fewer than four strong ratings and/or only one weak component. Weak studies have two or more weak components. Only one reviewer conducted quality assessments for these articles.

### Data synthesis/analysis

Implementation status and approaches and physical activity outcomes across each eligible study were summarized descriptively. The TDF was used to code the implementation barriers and facilitators reported by teachers, principals, and administration across the studies in order to identify what needs to change for behavior/implementation to change.

#### Reliability of method

Agreement of barrier and facilitator extraction by coders was assessed by percent agreement. To analyze the factors that influenced the implementation of DPA across studies, two researchers independently coded barriers and facilitators to the TDF domains in seven rounds. For each round, a percentage of the total extracted list of barriers and facilitators were randomly selected (across all papers). In the first round, the theoretical definitions of each TDF domain were used as a framework to guide coding. Coders met to discuss discrepancies after the first round (and every round thereafter), and a coding manual was refined to the context of our research topic for subsequent coding rounds (see 3rd column in Additional file 4). Ongoing discussion and refinement between rounds ensured that recoding previous items was not necessary. In the first round, 9.85% of the total identified barriers and facilitators (*n* = 20) were coded using the TDF domain and definitions [24] (see Additional file 4). In rounds 2 and 3, an additional 11.8% (*n* = 24) and 12.8% (*n* = 26) was coded, respectively. In round 4, an additional 19.7% (*n* = 40) was coded. In round 5, 14.8% (*n* = 30) more were coded, and in round 6, 16.3% (*n* = 33) was coded. In round 7, the last 14.8% (*n* = 30) was coded. Where coding varied, consensus was achieved through discussion after each round. Percent agreements, Cohen’s Kappa statistic [35] and prevalence adjusted bias adjusted Kappa statistic (i.e., PABAK) [36] were used to show agreement between coders for new items coded at each round. PABAK was used to account for the high prevalence of not assigning more than one domain to each barrier. Intercoder agreement values of 0.60–0.79 indicate “substantial” reliability, and those above 0.80 are “outstanding” [37]. Finally, main themes from barrier/facilitator coding were identified, and illustrative comments for each theme were selected.

## Results

### Characteristics of eligible studies

Selection of eligible studies is summarized in Fig. 1. The search resulted in 66 articles being retrieved and 38 being excluded for not meeting the eligibility criteria. Overall, a total of 15 articles and reports met the eligibility criteria for the current review [18–20, 38–49], ten of which examined barriers and facilitators to implementation [38, 40, 41, 43–49]. Of the 15 studies that met the inclusion criteria, 11 articles evaluated the Ontario DPA policy [19, 20, 38–46], and 2 articles were from both Alberta [47, 48] and British Columbia [18, 49]. Table 1 summarizes each study based on province, evaluation type, methods and data used, participants, evaluation indicators, and main findings. There were an equal number of quantitative (*n* = 6), qualitative (*n* = 5), and mixed methods (*n* = 4) studies included in this review. The majority of the studies evaluated implementation (*n* = 13), and two studies evaluated a combination of implementation by teachers and effectiveness on student’s physical activity levels.

**Table 1:** Summaries of daily physical activity policy evaluations in Canada

Author, year	Province	Evaluation type	Methods	Data source(s)	Study participants (*n* = sample size)	Evaluation indicators/questions	Main findings related to DPA
Patton, 2012	ON	Implement	QUANT	Survey	Teachers (*n* = 145)	% implementation, implementation approaches, teacher’s perspectives (supports and barriers, attitudes)	45% often or always conduct DPA on days with no PE; 85% report sufficient resources and 89% report sufficient knowledge; 46% think DPA should be more structured; 65% reported lack of monitoring; 60% support DPA
Patton et al. 2014	ON	Implement	QUANT	Survey	Students (*n* = 146)	Implementation approaches, barriers, attitudes	46% reported DPA every day there is not PE; barriers: student disruption, withholding DPA as punishment; majority of students agree that there is enough space/equipment/time to do DPA every fday and majority enjoy it
AGO, 2013	ON	Implement	MIXED	Survey, interviews, document review	School boards (teachers and principals) (*n* = unknown)	Procedures for implementing, monitoring and measurement and reporting of DPA in schools	Neither the Ministry or school boards are monitoring implementation; majority of principals reported students not getting DPA; barriers: lack of time and space, focus on literacy
Strampel et al. 2014	ON	Implement	MIXED	Survey (with open-ended questions)	Teachers (*n* = 137)	Barriers and possible solutions to DPA implementation	Barriers: lack of time, resources, space, and staff and student buy-in; possible solutions: new games with minimal equipment, more indoor DPA activities, better infrastructure, more resources, whole-school DPA approach, student leaders/DPA role models, school-community links for DPA
Robertson-Wilson and Lévesque, 2009	ON	Implement	QUAL	Archival documents	N/A	Framework used to examine implementation approaches and challenges	DPA policy accounts for several factors (allocation of resources, task specification) important for implementation but not all (sustainability of resources, policy value, evaluation plans)
Brown and Elliott, 2015	ON	Implement	QUAL	Semi-structured interviews	Teachers (*n* = 14) and principals (*n* = 5)	DPA implementation approaches, facilitators, barriers, perceived outcomes, and suggestions for change	Approaches: multiple breaks, student-led activities, integration into other subjects; facilitators: staff support, available resources, training sessions; barriers: lack of time, space, equipment, training, student motivation, and monitoring; outcomes: increased focus, enjoyment, classroom environment; suggestions: whole-community approach, more space, resources, and monitoring
Rickwood, 2015	ON	Implement	QUAL	Semi-structured interviews	Teachers (*n* = 5) and school administrators (*n* = 4)	Perceived barriers, association between beliefs about DPA policy and student PA levels	Barriers: diminishing priority of DPA, used as a behavior management strategy, lack of student motivation
Allison et al. 2014	ON	Implement	QUAL	Semi-structured group and individual interviews	Central players in development and implementation of DPA (*n* = 10)	Factors influencing development and implementation, roles of key players, barriers, and current status of DPA	Issues of flexibility and accountability; several relationships to assist with implementation; barriers of tight timeline, lack of support, insufficient training, lack of facilities, space and equipment, poor weather, increased teacher burden, lack of accountability; inconsistent implementation and lack of evaluation plan
Gilmore and Donohoe, 2016	ON	Implement	QUANT	Survey	Teachers (*n* = 136)	Implementation status; perceived competence, motivation and skills to deliver DPA	46% of teachers reported that DPA is not being delivered; majority of teachers lack competence, motivation and skills to deliver DPA
Stone et al. 2012	ON	Combination	QUANT	Accelerometer and classroom schedules	Students (*n* = 856)	Total PA, frequency of DPA schedule, and quality, number and duration of sustained bouts of MVPA (≥5 min), BMI	Less than 50% get DPA every day, but for those that do they are more active, more likely to meet guidelines and less likely to be overweight; no child engaged in sustained MVPA for ≥20 min
Hobin et al. 2010	ON	Combination	QUANT	Survey	Students (*n* = 2379) and school administrators (*n* = 30)	Student-level (sex, grade, #PE classes/week, MVPA minutes) and school-level (intramurals and interschool programs, DPA implementation model) characteristics	70% of schools offered DPA only on days without PE; student PA levels were associated with PE frequency but not DPA implementation model
Kennedy et al. 2010	AB	Implement	MIXED	Interview or survey	Principals/vice-principals (*n* = 55) and PE teachers (*n* = 7)	DPA knowledge, % implementation, approaches, barriers	100% principals and teachers reported full implementation; 80% of schools provided daily PE
Alberta Education, 2008	AB	Implement	MIXED	Survey	Principals (*n* = 387) and teachers (*n* = 638)	Resources and supports for DPA, PE, DPA activities, attitudes, challenges, monitoring status	Positive perceptions of DPA, higher for principals; multiple approaches for implementation and challenges (scheduling, lack of facilities/space); 60% of principals monitor DPA
Watts et al. 2014^a^	BC	Implement	QUANT	Survey	Principals (*n* = 351)	Environment changes; minutes of PE per week and delivery method of PE	≥150 min PE/week increased from 34.1 to 48.1% before and after implementation
Mâsse et al. 2013	BC	Implement	QUAL	Semi-structured Interviews	Principals (*n* = 17) and teachers (*n* = 33)	Perceived implementation, styles/change, factors that impeded or facilitated implementation of DPA	Perceived implementation varies between principals and teachers; prescriptive vs. non-prescriptive approach; major themes: relative advantage, compatibility, complexity, observability, facilitators (contextual factors)

*ON* Ontario, *AB* Alberta, *BC* British Columbia, *Implement* implementation evaluation, *QUANT* quantitative, *QUAL* qualitative, *MIXED*, mixed methods, study used both quantitative and qualitative measures, Combination evaluation type means study/report examined some aspect of implementation process and policy effectiveness; *AGO* Office of the Auditor General of Ontario, *PE* physical education, *MVPA* moderate-to-vigorous physical activity, *BMI* body mass index

^a^Study examined nutritional policy in middle and high school, only relevant data from grade 6 and DPA examined

### Study quality

Due to nature of a scoping review and the limited research available, articles were not excluded based on their quality rating (see Additional file 5 and 6). Both raters were in complete agreement of overall global ratings for process evaluations. While not excluded from the review, we were not able to assess the quality of the Auditor General’s Office report [40] due to poor reporting. Specifically, there was a lack of detail on the methods employed and interpretation of the results. Of the remaining studies evaluating the implementation of DPA, 8 studies received moderate process scores [18, 42–45, 47–49] and 4 studies received weak process scores [38, 39, 41, 46]. Based on the process that measures quality assessment criteria, no studies received strong process scores. This was most likely due to the lack of multiple data collection methods and the inability to measure data on multiple occasions. Only one study managed to include measurements before the DPA policy was implemented to measure the change in the school environment [18]. No studies measured policy outcomes related to implementation dose or quality (item P8). Based on the EPHPP quality assessment tool, the two effectiveness articles received weak global ratings, due to poor reporting because secondary data was presented (original articles were retrieved to assess methods) [50, 51]. Of note is that the tool is not specific to observational studies, so some items were not applicable.

### Barrier and facilitator extraction and coding reliability

Ten studies that reported factors that influence the implementation of DPA were included (see Fig. 1). The two independent coders extracted a total of 76 barriers/facilitators from three randomly selected articles, and percent agreement for barrier and facilitator extraction was 75.0%. Across each barrier and facilitator coding rounds, the average intercoder agreement was outstanding. The initial coding in round 1 showed substantial agreement levels, but reliability improved following refinement of the coding manual (see Table 2).

**Table 2:** Intercoder agreement statistics including percent agreement, Kappa and PABAK and the number of observations used during each coding round

Round	% total (*n* observations)	Mean percent positive agreement (*n* observations^a^)	Mean Kappa (±SD)	Mean PABAK (±SD)
Round 1	9.85 (20)	70.0 (20)	0.66 ± 0.50	0.90 ± 0.15
Round 2	11.8 (24)	88.5 (26)	0.90 ± 0.25	0.97 ± 0.08
Round 3	12.8 (26)	71.0 (31)	0.79 ± 0.41	0.94 ± 0.12
Round 4	19.7 (40)	76.2 (42)	0.74 ± 0.44	0.92 ± 0.12
Round 5	14.8 (30)	84.2 (38)	0.85 ± 0.35	0.94 ± 0.12
Round 6	16.3 (33)	77.5 (40)	0.83 ± 0.34	0.94 ± 0.11
Round 7	14.8 (30)	84.8 (33)	0.90 ± 0.29	0.97 ± 0.09

*Kappa* Cohen’s Kappa statistic [35], *PABAK* prevalence adjusted bias adjusted Kappa statistic [36]

^a^Some barriers were coded under multiple domains if applicable. Mean percent was calculated based on each code the BF was given

### Implementation status

While one study reported 100% successful implementation by principals and teachers in a sample of Calgary elementary schools [47], most studies revealed that schools are not meeting the implementation requirements. In their DPA study in Ontario, Stone and colleagues [20] categorized schools on a continuum according to implementation schedule: according to parents, 16% of students were occasionally (1–2 days per week), 34% of students were often (3–4 days per week), and 49% of students were always (5 days per week) given opportunities to be active each day for 20 min. In one school district in Ontario, only 45% of teachers and 46% of students reported always or often doing DPA on days with no physical education [38, 39]. In BC, Watts and colleagues [18] found that 65% of the schools they surveyed obtained full implementation of DPA, while another study revealed that principals perceived greater implementation (90%) compared to teachers (43%) [49].

### Implementation approaches

Implementation approaches used by DPA deliverers to fulfill DPA requirements included many different approaches. In BC, Mâsse, Naiman, and Naylor [49] categorized implementation style taken by schools as either prescriptive or non-prescriptive. Prescriptive approaches require all children to participate during instructional time while non-prescriptive approaches provide children with more opportunities to be active during non-instructional time. The majority of elementary schools across each province adopted a prescriptive approach by increasing physical education classes during the week [18, 43, 48] or scheduling DPA activity class into the timetable [43, 47–49]. Ontario schools used some creative methods to deliver DPA during instructional time, including integrating DPA into other curriculum subjects, taking multiple smaller breaks throughout the day and allowing older students to lead DPA activities for younger classes [43]. Non-prescriptive approaches included providing more opportunities and access to facilities at recess and lunch breaks, without providing additional times to be active during instructional time [40, 47–49]. For example, in Alberta, 57% of schools reported increasing resources through the purchasing of equipment for gym and recess [48]*.*

### Identified barriers and facilitators

A total of 203 barriers/facilitators were extracted across the ten studies. Table 3 outlines the number of barriers/facilitators that were identified across DPA studies based on the TDF domains. Some of these barriers were coded under multiple domains, resulting in a total of 230 coded barriers/facilitators. The most commonly coded TDF domains were *environmental context and resources (*ECR; *n* = 86; 37.4%), *beliefs about consequences* (*n* = 41; 17.8%), and *social influences* (*n* = 36; 15.7%). No barriers/facilitators were coded in *memory*, *attention and decision processes*, *goals*, or *optimism* domains. Only four of the ten articles that examined implementation used theory to guide the study. Identified themes from the TDF domains are listed in Additional file 7.

**Table 3:** TDF identified barriers and facilitators of DPA

Paper (author, year)	Province	Participants	Method	Scale	Theory	Total BFs identified (*n*)	TDF barriers (*n*)	Comments
Mâsse et al. 2013	BC	Principals and teachers	Interviews	N/A	DOI	24	ECR (9) Beliefs about consequences (4) SPRI (3) Social influences (2) Skills (2) Beliefs about capabilities (2) Knowledge (2)	Theory was used to arrange study findings, but did not guide interview.
Kennedy et al. 2010	AB	Principals, vice-principals, and PE teachers	Survey	Check all that apply	N/A	12	ECR (8) Social influences (4) Skills (1) Knowledge (1)	The survey contained preset answers; participants were allowed to give more than one answer. Frequencies (%) were reported, and factors were extracted if at least 50% of the respondents checked that the barrier was present.
Strampel et al. 2014	ON	Teachers	Survey	Likert scale (1 = strongly disagree to 5 = strongly agree)	N/A	13	ECR (8) Social influences (3) Beliefs about capabilities (1) SPRI (1) Skills (1) Knowledge (1)	Frequencies, means and standard deviations were reported. Extraction and coding was based off frequencies. The middle anchor was “neither agree nor disagree” and any responses for this option were not included in determining if the factor was extracted. Some items were reverse scored, and therefore, these were accounted for in item extraction. All open-ended responses were extracted.
Patton, 2012	ON	Teachers	Survey	Likert scale (1 = never to 5 = always)	N/A	14	Beliefs about consequences (6) ECR (4) Social Influences (2) Emotion (1) Reinforcement (1) Intentions (1)	Only extracted barriers that at least 50% of respondents believed sometimes, often, or always influenced delivery of DPA.
Allison et al. 2014	ON	Key informants (involved in initial development and implementation of DPA)	Interviews	N/A	N/A	24	ECR (13) Beliefs about consequences (3) Skills (3) Knowledge (3) Reinforcement (3) SPRI (2) Social Influences (2) Intentions (1) Beliefs about capabilities (1)	
Brown and Elliot, 2015	ON	Teachers and principals	Interviews	N/A	SET and ANGELO	61	ECR (22) Beliefs about consequences (13) Social Influences (13) Skills (6) Reinforcement (5) Intentions (3) Beliefs about capabilities (3) Knowledge (3) SPRI (1) Behavioral regulation (1)	
Rickwood, 2015	ON	Teachers and administrators	Interviews	N/A	CST	15	ECR (5) Beliefs about consequences (4) Social influences (3) Intentions (1) Beliefs about capabilities (1) SPRI (1)	Participants discussed barriers more in relation to PE, coaching, and overall general PA; not always DPA-specific. However, DPA policies do include PE as a method to meet DPA guidelines, and therefore, all reported barriers and facilitators were extracted.
Alberta Education, 2008	AB	Principals and teachers	Survey	Likert scale (1 = strongly agree to 5 = strongly disagree)	N/A	33	ECR (13) Beliefs about consequences (11) Social influences (7) Beliefs about capabilities (2) Skills (1) Knowledge (2) SPRI (1)	Only extracted barriers that received at least 50% agreement (somewhat agree, strongly agree). The middle anchor was “neither agree nor disagree” and any responses for this option were not included in determining if the factor was extracted. Principals reported less challenges associated with DPA implementation and perceived more positive outcomes than teachers. Despite this difference, the same extraction criteria applied irrespective of whether it was the teachers or principals agreeing/disagreeing that the factor was present.
Auditor General’s Office, 2013	ON	School boards (principals and teachers)	Surveys, interviews, document review	Not reported	N/A	3	ECR (3)	Survey question type was not reported. Descriptive results were presented on the most influential barriers. These factors were extracted.
Gilmore and Donohoe, 2016	ON	Teachers	Survey	Likert scale (7-pt scale from strongly disagree to strongly agree; anchors not provided)	FMST	4	Skills (2) ECR (1) Knowledge (1) Beliefs about capabilities (1) Intentions (1)	Only extracted barriers that received at least 50% agreement (agree, strongly agree). The middle anchor was “neither agree nor disagree” and any responses for this option were not included in determining if the factor was extracted.

*BC* British Columbia, *AB* Alberta, *ON* Ontario, *PE* physical education, *DPA* daily physical activity policy, *PA* physical activity, *DOI* diffusion of innovations, *SET* Social Ecological Theory, *ANGELO* Analysis Grid for Environments Linked to Obesity Framework, *CST* Cultural Systems Theory, *FMST* Ford’s Motivation Systems Theory, *TDF* Theoretical Domains Framework, *ECR* environmental context and resources, *SPRI* social/professional role and identity, *N/A* not applicable

### Effectiveness of DPA policy implementation on children’s physical activity

Only 2 of the 15 articles examined the impact of DPA on student’s physical activity behavior [18, 19]. Hobin and colleagues [19] examined associations between student self-reported MVPA and schools’ DPA implementation model and found that student physical activity was associated with PE frequency per week but not the DPA implementation model (i.e., DPA only on days without PE, in addition to daily PE or as part of daily PE). Stone and colleagues [20] used accelerometers and classroom schedules to compare total physical activity and sustained bouts of MVPA to frequency of DPA schedule. They found that less than 50% of students received DPA every day, and no child engaged in sustained MVPA for 20 min as required by the DPA guidelines. However, for children who did receive DPA every day, they were more active overall, more likely to meet PA guidelines, and less likely to be overweight compared to students who did not receive DPA.

## Discussion

With the limited research examining the DPA policy in Canada, the current status and approaches used to implement DPA, and the impact on student’s physical activity levels is not well understood; however, this review revealed that DPA deliverers (i.e., teachers, principals, administration) often report many barriers to DPA implementation, most of which relate to the environmental context and resources (i.e., lack of training, time, and resources), beliefs about consequences (i.e., burden on teacher, classroom influences), and social influences (i.e., lack of student/parent interest) domains of the TDF. Understanding these implementation barriers from a theoretical perspective is the key to creating solutions to overcoming them in the future. Our review adds this theoretical analysis to the existing literature and is relevant to other studies examining the implementation of school-based interventions and polices that commonly report similar barriers and facilitators to uptake [32].

### Barriers and facilitators and theoretically informed solutions to DPA implementation

Nearly all implementation evaluations reviewed for this article examined staff member’s perspectives regarding the barriers and facilitators to DPA policy implementation. Common themes emerged irrespective of province, context/scheduling requirement (i.e., instructional or non-instructional), or data collection methodology (i.e., quantitative or qualitative), and the majority of barriers reported by teachers and principals related to the TDF theoretical domains of ECR, social influences, and beliefs about consequences. These implementation barriers experienced by DPA deliverers are similar to those reported by others implementing similar school-based PA policies [52–56], highlighting that school policy implementers experience similar barriers and challenges when implementing PA initiatives in a school context.

A primary strength of this study as compared to previous reviews is that in using a theoretical framework to understand policy implementation, researchers can develop theoretically informed solutions to the identified barriers and design interventions that can better target these problems in the future [57]. A TDF analysis provides the behavioral diagnosis of what needs to change in a specific context in order for a target behavior to occur and can be linked to intervention functions and techniques to change behavior through guidance of the Behaviour Change Wheel framework (BCW) [22]. This review highlights the need to create interventions that target barriers relating to the (1) ECR, (2) beliefs about consequences, and (3) social influences domains. Intervention functions that have been linked to these domains include: (1) training, restriction, environmental restructuring and enablement. (2) Education, persuasion, and modeling, and (3) restriction, environmental restructuring, modeling and enablement, respectively [22]. Therefore, DPA implementation may improve if some or all of these intervention functions are directed at the DPA deliverers through interventions. For example, one strategy to overcome the commonly reported barrier of lack of training (coded in the TDF domains ECR, *skills*, and *knowledge*) would be for Ministries of Education and/or school boards to provide additional and ongoing training to teachers on how to conduct DPA during the instructional and non-instructional school day. Similarly, to target teachers’ perception of a lack of time (i.e., ECR) and to minimize the burden that they feel about fitting DPA during the busy school day (i.e., beliefs about consequences), school boards can emphasize how DPA positively benefits children’s focus and concentration (i.e., education) or require that DPA is a part of the overall curriculum and monitor it more readily (i.e., environmental restructuring). Focusing specifically on teacher’s reported implementation barriers and perceptions will assist with policy implementation, considering that they express less support, perceive less effectiveness of, and report more barriers for DPA implementation than principals [38, 43, 48].

### Low adoption of DPA implementation

The level of perceived implementation adoption is inconsistent across the three provinces. Overall, it appears that only about half of the elementary schools studied are meeting their respective DPA time requirement, as self-reported by teachers and principals. However “[the] self-reported findings may reflect what is scheduled versus actual policy implementation” (p.S75) as made evident by direct observations in a school-based PA policy evaluation in Alabama [58]. Moreover, scheduling DPA into the school day provides children with the *opportunity* to be active, but does not guarantee that students are active during this time.

### Implementation approaches

Implementation approaches across Canada have varied, with the majority of schools adopting prescriptive (e.g., additional PE and scheduling DPA into timetable, integrating DPA into other curriculum subjects, taking multiple smaller breaks throughout the day) approaches, and some schools are using non-prescriptive (e.g., intramurals, lunch hour games and open access to facilities and equipment) approaches (defined by Mâsse and colleagues [49]). Non-prescriptive approaches would allow schools and teachers to take a more *hands-off* approach and possibly minimize the two major perceived barriers relating to ECR, including a lack of time in schedule [38, 40, 41, 43, 47–49] and conflicting with other curricular demands [38, 40, 41, 43, 45, 49]. Unfortunately, the implementation delivery methods currently used are not linked to PA outcomes, and as such, it is unknown how effective these specific approaches are at increasing children’s physical activity levels at school. A more specific examination of the behavior change techniques [59] that teachers, principals, and administrative staff use to deliver DPA would be beneficial for linking implementation approaches to identified barriers, and ultimately, PA outcomes.

### Future research

There is an obvious need for future evaluation to examine DPA policy implementation and effectiveness across all three provinces. Few studies have evaluated the effectiveness of the various DPA implementation approaches employed by elementary schools on student’s PA levels. To understand the impact of these policies, further research that uses objective measures of PA in children is needed. Even though DPA policy implementation barriers and facilitators have been examined in depth, it is unclear whether or not these findings have been utilized to change implementation practices. In particular, it is unclear if and what strategies have been provided to or used by schools to overcome barriers and facilitate implementation of the policy. In order for the DPA policy to meet prescribed outcomes, it is essential that current evaluation research findings be translated into usable forms to allow for schools to adopt implementation procedures according to research-based evidence. The use of the TDF to analyze barriers and facilitators to implementation assists with this process for future research interventions.

### Strengths and limitations

While the strength of this review is the utilization of a theoretical framework to categorize the factors that influence the implementation of the DPA policy across three Canadian provinces, it is important to recognize its limitations. A limited number of databases were searched and therefore our search for articles was not exhaustive. It is possible that the search terms did not result in the complete retrieval of DPA policy articles in this context. The exclusion of dissertation data may also have limited relevant research from this review. Future research should consider a formal systematic review that includes similar DPA policies from international jurisdictions to provide more comprehensive and more generalizable findings.

Only one author screened articles for eligibility and extracted data from all studies. Of the studies that were included, it is difficult to compare findings and therefore draw conclusions from this review, due to the nature of heterogeneity in policy implementation and evaluation. Barriers and facilitators were not always explicitly discussed, and the authors did not have access to the raw data from each eligible article. Therefore only barriers and facilitators that were reported by the original authors could be extracted and coded, and findings may not encompass the full range of factors that influence DPA implementation. Given the heterogeneity of reporting barriers and facilitators across studies, we found it useful to code the barriers and facilitators in rounds, using the TDF domain definitions. After each round, consensus discussion allowed us to refine the coding manual to the context of the research topic, and this strengthened our agreement.

Our parameters for barrier and facilitator extraction excluded factors that may have a significant role on implementation. Even if most respondents did not agree that a barrier or facilitator influenced implementation, it still represents a factor that should be considered in tailoring interventions. However, while some factors may not have been extracted from one study, they may have been extracted from other studies and therefore were still captured in our findings. In the future, it would be helpful for authors to use consistent methods for measuring and reporting barriers and facilitators (e.g., using a theoretical framework like the TDF). Finally, the level at which the barrier/facilitator was being discussed in the original research was not always clear (i.e., does the factor affect the teacher implementing DPA or the student engaging in physical activity?). The use of the TDF allowed us to accomplish this by categorizing the barriers/facilitators according to the DPA deliverer (i.e., teacher, principal, administration); however, it is possible that the level at which the barrier/facilitator was working was incorrectly interpreted by the researchers.

## Conclusions

Overall, the research evaluating the daily physical activity policies in Ontario, Alberta, and British Columbia has many shortcomings. Of particular concern is the lack of evaluation in British Columbia and Alberta. While the majority of studies have examined the process of DPA policy implementation in elementary schools, a lack of implementation adoption undermines future evaluation of the policy’s effectiveness on student PA levels. Only when schools report greater adherence to implementation, will there be value in measuring the policy’s effectiveness. Also, “[b]ecause policy and program implementation are evolving processes that typically entail extensive adaptation, evaluation efforts must continue to attend to process issues” (p. 56) [12]. Important process issues include addressing the barriers to implementation. While research evidence is limited and the use of theory to guide our understanding of policy evaluation has been scarcely utilized, this review provides a theoretical lens in which to understand the barriers and facilitators to DPA policy implementation. It is our hope that this analysis will assist researchers in creating interventions to overcome implementation barriers and more successfully fulfill policy guidelines to be able to evaluate the effectiveness of these policies on student’s PA levels in the future.

## Additional files

Additional file 1: PRISMA Checklist. Completed PRISMA checklist indicating page number in manuscript of relevant content. (DOC 58 kb)

Additional file 2: Process measures quality assessment. Quality assessment criteria for process evaluations, adapted from Wierenga and colleagues [33]. (DOCX 73 kb)

Additional file 3: Impact measures quality assessment. Quality assessment criteria for effectiveness evaluations, adapted from Thomas and colleagues [34]. (DOCX 81 kb)

Additional file 4: TDF coding manual. TDF domains and definitions used to code barriers and facilitators. (DOCX 127 kb)

Additional file 5: Quality assessment of implementation studies. Quality ratings for each implementation study using Wierenga and colleagues [33] quality assessment criteria. (DOCX 86 kb)

Additional file 6: Quality assessment of effectiveness studies. Quality ratings for each effectiveness study using Thomas and colleagues [34] quality assessment criteria. (DOCX 53 kb)

Additional file 7: Themed barriers and facilitators to DPA implementation by theoretical domain. Identified themes to implementation barriers and facilitators arranged by TDF domains. (DOCX 132 kb)
