# Supplementary material for: A theoretical analysis of the barriers and facilitators to the implementation of school-based physical activity policies in Canada: a mixed methods scoping review
Source: Implement Sci. 2017 Mar 27;12:41. doi: 10.1186/s13012-017-0570-3 (PMC5369225; doi:10.1186/s13012-017-0570-3)
Supplement: Supplementary file 2 — Process measures quality assessment. Quality assessment criteria for process evaluations, adapted from Wierenga and colleagues [33]. (DOCX 73 kb) [file 13012_2017_570_MOESM2_ESM.docx]

**Additional File 2. Process measures quality assessment***

| **Item** | **Evaluation Criteria** |
| --- | --- |
| P1) Level of evaluation | Positive if implementation was evaluated on 2 or more levels (e.g., student/family, teacher) |
| P2) Definition of process variables | Positive if process variables were adequately described |
| P3) Process variables | Positive if four or more process variables were reported |
| P4) Data collection | Positive if two or more techniques were used (i.e., triangulation) |
| P5) Timing of data collection | Positive if process variables were measured on multiple occasions (e.g., pre, during and/or post implementation) |
| P6) Quantitative process variables | Positive if quantitative process outcomes were assessed using methods of acceptable quality |
| P7) Qualitative process variables | Positive if qualitative study design was adequately described (e.g., participant selection, setting, data collection) |
| P8) Outcome related to implementation | Positive if outcomes were evaluated in the context of implementation dose/quality |

*Process measures quality assessment adapted from Wierenga et al., 2013
