# Supplementary material for: A theoretical analysis of the barriers and facilitators to the implementation of school-based physical activity policies in Canada: a mixed methods scoping review
Source: Implement Sci. 2017 Mar 27;12:41. doi: 10.1186/s13012-017-0570-3 (PMC5369225; doi:10.1186/s13012-017-0570-3)
Supplement: Supplementary file 3 — Impact measures quality assessment. Quality assessment criteria for effectiveness evaluations, adapted from Thomas and colleagues [34]. (DOCX 81 kb) [file 13012_2017_570_MOESM3_ESM.docx]

**Additional File 3. Impact measures quality assessment ***

| **Item** | **Evaluation Criteria** |
| --- | --- |
| Selection bias | Are the individuals selected to participate in the study likely to be representative of the target population?  What percentage of selected individuals agreed to participate? |
| Study design | Was the study described as randomized?  If yes, was the method of randomization described?  If yes, was the method appropriate? |
| Confounders | Were there important differences between groups prior to the intervention?  If yes, indicate the percentage of relevant confounders that were controlled either in the design (e.g., stratification, matching) or analysis. |
| Blinding | Was (were) the outcome assessor(s) aware of the intervention or exposure status of participants?  Were the study participants aware of the research question? |
| Data collection methods | Were data collection tools shown to be valid?  Were data collection tools shown to be reliable? |
| Withdrawals and dropouts | Were withdrawals and dropouts reported in terms of numbers and/or reasons per group?  Indicate the percentage of participants completing the study |

*Quantitative quality assessment performed using the quality assessment tool for quantitative studies developed by the Effective Public Health Practice Project (EPHPP; Thomas et al., 2004)
