# Supplementary material for: A theoretical analysis of the barriers and facilitators to the implementation of school-based physical activity policies in Canada: a mixed methods scoping review
Source: Implement Sci. 2017 Mar 27;12:41. doi: 10.1186/s13012-017-0570-3 (PMC5369225; doi:10.1186/s13012-017-0570-3)
Supplement: Supplementary file 4 — TDF coding manual. TDF domains and definitions used to code barriers and facilitators. (DOCX 127 kb) [file 13012_2017_570_MOESM4_ESM.docx]

**Additional File 4. TDF coding manual**

| **TDF Domain** | **Definition** | **Notes (Themes)** |
| --- | --- | --- |
| Skills (physical, cognitive and interpersonal) | An ability or proficiency acquired through practice | - Lack of training* |
|  |  |  |
| Knowledge | An awareness of the existence of something | - Lack of training* |
| Memory, attention and decision Processes | The ability to retain information, focus selectively on aspects of the environment and choose between two or more alternatives |  |
| Behavioural regulation | Anything aimed at managing or changing objectively observed or measured actions |  |
| Social/professional role and identity (SPRI) | A coherent set of behaviours and displayed personal qualities of an individual in a social or work setting |  |
| Beliefs about capabilities | Acceptance of the truth, reality, or validity about an ability, talent, or facility that a person can put to constructive use |  |
| Optimism | The confidence that things will happen for the best or that desired goals will be attained |  |
| Beliefs about consequences | Acceptance of the truth, reality, or validity about outcomes of a behaviour in a given situation | - Child enjoyment/fun - Impact on child learning |
| Intentions | A conscious decision to perform a behaviour or a resolve to act in a certain way | - Priority at individual level |
| Goals | Mental representations of outcomes or end states that an individual wants to achieve |  |
| Reinforcement | Increasing the probability of a response by arranging a dependent relationship, or contingency, between the response and a given stimulus |  |
| Emotion | A complex reaction pattern, involving experiential, behavioural, and physiological elements, by which the individual attempts to deal with a personally significant matter or event | - Personal fun/enjoyment |
| Environmental context and resources (ECR) | Any circumstance of a person's situation or environment that discourages or encourages the development of skills and abilities, independence, social competence, and adaptive behaviour | - Lack of training* - Autonomy supportive - Curriculum demands |
| Social influences | Those interpersonal processes that can cause individuals to change their thoughts, feelings, or behaviours | - Supportive others - Mentorship - Priority at group level - Generic support (no resources specified) |

Coding manual based on definitions provided in Cane, O’Connor & Michie (2012). TDF, Theoretical Domains Framework

*A general lack of training was always coded under Skills AND Knowledge AND ECR.
