# Supplementary material for: A theoretical analysis of the barriers and facilitators to the implementation of school-based physical activity policies in Canada: a mixed methods scoping review
Source: Implement Sci. 2017 Mar 27;12:41. doi: 10.1186/s13012-017-0570-3 (PMC5369225; doi:10.1186/s13012-017-0570-3)
Supplement: Supplementary file 5 — Quality assessment of implementation studies. Quality ratings for each implementation study using Wierenga and colleagues [33] quality assessment criteria. (DOCX 86 kb) [file 13012_2017_570_MOESM5_ESM.docx]

**Additional File 5. Quality assessment of implementation studies***

|  | Process measures quality assessment* | | | | | | | | |
| --- | --- | --- | --- | --- | --- | --- | --- | --- | --- |
|  | P1 | P2 | P3 | P4 | P5 | P6 | P7 | P8 | Global process rating |
| Kennedy et al., 2010 | + | + | + | − | − | − | N/A | N/A | Moderate |
| Robertson-Wilson & Lévesque, 2009 | − | + | + | − | − | N/A | + | N/A | Moderate |
| Strampel et al., 2014 | − | + | − | + | − | − | − | N/A | Weak |
| Patton, 2012 | − | + | + | − | − | − | N/A | N/A | Weak |
| Patton et al., 2014 | − | − | − | − | − | − | N/A | N/A | Weak |
| Allison et al., 2014 | + | + | + | − | − | N/A | + | N/A | Moderate |
| Brown & Elliot, 2015 | + | + | + | − | − | N/A | + | N/A | Moderate |
| Rickwood, 2015 | + | − | + | − | − | N/A | + | N/A | Moderate |
| Gilmore & Donohoe, 2016 | − | − | + | − | − | − | N/A | N/A | Weak |
| Alberta Education, 2008 | + | + | + | − | − | + | N/A | N/A | Moderate |
| Mâsse et al., 2013 | + | + | + | − | − | N/A | + | N/A | Moderate |
| Watts et al., 2014 | − | + | + | − | + | + | N/A | N/A | Moderate |

*Process measures quality assessment adapted from Wierenga et al (2013). See Additional file 2 for definitions of P1-P8
