# Supplementary material for: A theoretical analysis of the barriers and facilitators to the implementation of school-based physical activity policies in Canada: a mixed methods scoping review
Source: Implement Sci. 2017 Mar 27;12:41. doi: 10.1186/s13012-017-0570-3 (PMC5369225; doi:10.1186/s13012-017-0570-3)
Supplement: Supplementary file 6 — Quality assessment of effectiveness studies. Quality ratings for each effectiveness study using Thomas and colleagues [34] quality assessment criteria. (DOCX 53 kb) [file 13012_2017_570_MOESM6_ESM.docx]

**Additional File 6. Quality assessment ratings for effectiveness studies**

|  | Quantitative quality assessment* | | | | | | |
| --- | --- | --- | --- | --- | --- | --- | --- |
|  | Selection bias | Study design | Confounders | Blinding | Data collection methods | Withdrawals/  dropouts | Global quantitative rating |
| Stone et al., 2012^a^ | Moderate | Weak | N/A | Weak | Strong | Weak | Weak |
| Hobin et al., 2010^b^ | Weak | Weak | N/A | Weak | Moderate | N/A | Weak |

*Quantitative quality assessment performed using the quality assessment tool for quantitative studies developed by the Effective Public Health Practice Project (EPHPP; Thomas et al., 2004). See Additional file 3 for questions related to each component

^a^ Components reported in original study by Stone, Faulkner, & Buliung (2013)

^b^ Components reported in original study by Leatherdale, Manske, Faulkner, Arbour, & Bredin (2010)
