# Supplementary material for: A theoretical analysis of the barriers and facilitators to the implementation of school-based physical activity policies in Canada: a mixed methods scoping review
Source: Implement Sci. 2017 Mar 27;12:41. doi: 10.1186/s13012-017-0570-3 (PMC5369225; doi:10.1186/s13012-017-0570-3)
Supplement: Supplementary file 7 — Themed barriers and facilitators to DPA implementation by theoretical domain. Identified themes to implementation barriers and facilitators arranged by TDF domains. (DOCX 132 kb) [file 13012_2017_570_MOESM7_ESM.docx]

**Additional File 7. Themed barriers and facilitators to DPA implementation by theoretical domain**

| **TDF Domain^a^** | **Theme - Barriers** | **Theme - Facilitators** |
| --- | --- | --- |
| Skills | - Lack skills and experience in physical activity delivery - Lack of training^b^ | - Training opportunities |
| Knowledge | - Lack of training^b^ - Lack of direction in policy guidelines (i.e., uncertainty about what qualifies) | - Teacher experience, knowledge and comfort in physical activity delivery |
| Behavioural regulation |  | - Putting DPA in schedule |
| Social/professional role and identity (SPRI) | - Belief that DPA should be a shared responsibility (of school, parent and student) | - Policy compatible with school and teacher priorities |
| Beliefs about capabilities | - Difficulty in fitting DPA into curriculum - Teacher uncomfortable conducting DPA |  |
| Beliefs about consequences | - Burden on teacher (i.e., increased workload, responsibility, stress) - Classroom influences (i.e., noise, disruption, time away from other subjects) - Safety issues | - Student influences (i.e., child enjoyment/fun, leadership opportunities) - Impact on child learning (i.e., increased focus and attention) - Classroom influences (i.e., improves overall environment) |
| Intentions | - No motivation of teachers - Diminishing priority (compared to other subjects) | - Priority of schools and teachers - Teachers’ values (i.e., physical activity is important) |
| Reinforcement | - Lack of monitoring/accountability |  |
| Emotion |  | - Personal fun/enjoyment |
| Environmental context and resources (ECR) | - Lack of training^b^ - Lack of time in schedule - Competing academic curricular demands - Lack of resources (i.e., space/facilities, equipment) - Inclement weather - Unclear policy guidelines | - Adequate indoor and outdoor facility space - Provincially-made available resources and ideas - Access to a PE specialist |
| Social influences | - Lack of student/parent interest - Negative student outlook on physical activity | - Leadership and support from staff, administration and other school champions |

TDF, Theoretical Domains Framework; PE, physical education

^a^No BFs were coded to *Memory, attention and decision processes*, *Goals*, or *Optimism* domains

^b^A general lack of training was always coded under Skills AND Knowledge AND ECR.
